# The Eight-Chop Technique in Nonagenarian Patients: Real-World Intraoperative and Postoperative Outcomes

**DOI:** 10.3390/jcm15176737

**Published:** 2026-08-30

**Authors:** Tsuyoshi Sato

**Affiliations:** Department of Ophthalmology, Sato Eye Clinic, 3-3 Nemoto, Matsudo-Shi 271-0077, Chiba-Ken, Japan; perfect-eightchop@sato-ganka.com

**Keywords:** cataract surgery, corneal endothelial cell density, Eight-Chop Technique, nonagenarian, phacoemulsification

## Abstract

**Objectives**: To describe intraoperative and postoperative outcomes of the Eight-Chop Technique in elderly patients, with particular attention to nonagenarian patients undergoing cataract surgery. **Methods**: This retrospective study included consecutive eligible eyes undergoing Eight-Chop phacoemulsification and posterior chamber intraocular lens implantation at a single clinic. Eyes were stratified by age at surgery into 70–79 years, 80–89 years, and ≥90 years groups. Sixty eyes were included in each younger group, and all 49 eligible nonagenarian eyes were included. Eyes were not intentionally excluded from the younger groups because of poor pupillary dilation, zonular weakness, white cataract, pseudoexfoliation, glaucoma, age-related macular degeneration, or advanced nuclear grade. Preoperative characteristics, intraoperative parameters, best-corrected visual acuity (BCVA), intraocular pressure, and corneal endothelial cell density (CECD) were evaluated. Linear mixed-effects models accounted for fellow-eye correlation. **Results**: A total of 169 eyes were analyzed. The ≥90-year cohort had greater baseline complexity; intraoperative differences were descriptive, with the highest operative time, phacoemulsification time, aspiration time, cumulative dissipated energy, and irrigation fluid volume (all *p* < 0.001). Postoperative BCVA improved from baseline in all groups. Mean CECD loss at 19 weeks was −1.3 ± 5.7%, −0.7 ± 5.7%, and 3.5 ± 9.2% in the 70–79-, 80–89-, and ≥90-year groups, respectively. No serious intraoperative complications occurred. **Conclusions**: The Eight-Chop Technique was used across elderly age groups, including nonagenarian patients. Observed age-group differences represent associations in this retrospective dataset and do not establish causal age effects or comparative superiority, safety, or endothelial protection of the technique.

## 1. Introduction

As populations age, cataract is an increasingly important cause of reversible visual impairment in older adults, and the demand for cataract surgery among nonagenarians is expected to grow further [1,2,3,4]. Previous studies have demonstrated that phacoemulsification is generally effective and safe in patients over 90 years of age; however, these eyes may have a higher burden of systemic comorbidities and ocular fragility, which can influence perioperative management and surgical risk [3,4,5].

Despite acceptable visual outcomes, cataract surgery in very elderly patients, including nonagenarians, can be technically challenging because of dense nuclei, zonular weakness, shallow anterior chambers, and coexisting corneal endothelial compromise [6,7]. Intraoperative parameters such as ultrasound energy, operative time, and irrigation fluid volume are closely related to corneal endothelial cell loss and postoperative corneal edema after phacoemulsification [8,9,10,11]. However, detailed intraoperative performance data in nonagenarian patients remain limited.

The Eight-Chop Technique is a nucleus fragmentation method in which the lens is mechanically divided into eight small segments before phacoemulsification and aspiration [12]. By separating nuclear division from ultrasound application and allowing emulsification of small prefragmented pieces at the iris plane, this technique has been reported to improve surgical efficiency while reducing intraocular stress, ultrasound energy delivery, and fluidic load compared with conventional techniques such as divide-and-conquer or phaco-chop [12,13].

However, the intraoperative and postoperative outcomes of the Eight-Chop Technique specifically in nonagenarian patients have not been fully characterized. In addition, few studies have systematically compared intraoperative performance, ultrasound energy use, irrigation fluid volume, and corneal endothelial cell loss across different age groups using a unified surgical technique. Therefore, this study aimed to describe real-world intraoperative performance and postoperative visual and corneal endothelial outcomes of the Eight-Chop Technique in nonagenarian patients, in comparison with younger age-stratified reference cohorts. Eyes aged 70–79 and 80–89 years were included to provide clinical context rather than as directly comparable control groups. Although the same prespecified eligibility criteria were applied across age groups, the nonagenarian cohort had greater baseline clinical and surgical complexity. Therefore, comparisons among age groups were intended to be descriptive and were not designed to estimate the independent causal effect of age.

## 2. Materials and Methods

### 2.1. Study Design and Patients

This retrospective observational study included eyes of patients with age-related cataract who underwent phacoemulsification and posterior chamber intraocular lens implantation using the Eight-Chop Technique at a single clinic. Eligible eyes were identified from cases performed between 1 May 2021 and 31 December 2025 and were stratified according to age at surgery. The same prespecified inclusion and exclusion criteria were applied across all age groups. All consecutive eligible eyes aged 90 years or older were included. Sixty eligible eyes were randomly selected from each of the 70–79-year and 80–89-year age groups from cases treated during the same study period. All enrolled eyes were followed for a predefined 19-week postoperative observation period, which was completed by 30 June 2026 for the last case.

The enrolled eyes were stratified into three age groups according to age at surgery: 70–79 years, 80–89 years, and 90 years or older. The study aimed to describe intraoperative performance and postoperative outcomes of the Eight-Chop Technique across these age-stratified elderly cohorts, with particular attention to outcomes in nonagenarian patients.

Eyes were not intentionally excluded on the basis of poor pupillary dilation, obvious zonular weakness, white cataract, pseudoexfoliation, glaucoma, age-related macular degeneration, or Emery nuclear grade. These characteristics were recorded as part of the clinical and surgical profile of each cohort.

All three age groups were intended to reflect the real-world clinical spectrum of patients undergoing cataract surgery within their respective age ranges. Some postoperative visual-acuity data were missing because longitudinal assessment could not be completed in all cases. Because this was a retrospective observational study, baseline clinical characteristics and surgical complexity could still differ among age groups. Therefore, between-group comparisons were interpreted as observational associations and not as effects attributable to age alone.

To assess overlap with previous publications from our institution, cases were cross-checked using patient identifier, operated eye, and date of surgery. Cases that overlapped with previous reports were retained because the present study had substantively distinct primary outcomes, research objectives, and a comparative framework.

This study adhered to the tenets of the Declaration of Helsinki and was approved by the Institutional Review Board of Sato Eye Clinic (approval number 2021040102). Informed consent for surgery and use of clinical data for research was obtained from each patient, and retrospective data extraction and analysis for this manuscript were conducted only after ethics approval had been obtained.

### 2.2. Preoperative and Postoperative Examinations

Preoperatively, all patients underwent routine ophthalmic examinations including slitlamp biomicroscopy, fundus examination, measurement of best corrected visual acuity (BCVA), and intraocular pressure (IOP). BCVA was converted to logarithm of the minimum angle of resolution (logMAR) units for statistical analysis. Corneal endothelial parameters, including corneal endothelial cell density (CECD; cells/mm^2^), were measured using a noncontact specular microscope (EM-3000, Topcon, Tokyo, Japan). Nuclear firmness was graded according to the Emery nuclear grade [14].

Postoperative follow-up examinations were performed according to the routine clinical schedule. In the present study, data obtained at postoperative weeks 7 and 19 were used for analysis. The postoperative outcome measures included BCVA, IOP, and CECD. The percentage of CECD loss at 19 weeks was calculated as follows:

CECD loss (%) = (preoperative CECD − postoperative CECD at 19 weeks)/preoperative CECD × 100

### 2.3. Surgical Technique

All surgeries were performed by the same experienced surgeon using the Eight-Chop Technique with the Centurion phacoemulsification system (Alcon Laboratories, Fort Worth, TX, USA). A temporal clear corneal incision was created, followed by continuous curvilinear capsulorrhexis, hydrodissection, phacoemulsification, cortical aspiration, and implantation of a foldable posterior chamber intraocular lens in the capsular bag. The lens nucleus was mechanically divided using the Eight-Chop Technique and then emulsified and aspirated.

The details of the Eight-Chop Technique and the dedicated chopping instruments have been described previously [12]. In brief, before phacoaspiration, the nucleus was mechanically divided into eight small fragments. This approach has been proposed to facilitate nuclear removal by prefragmenting the nucleus before phacoaspiration. In the present study, its comparative effects on phacoemulsification energy, intraocular manipulation, or corneal endothelial outcomes were not evaluated. Standard phacoemulsification settings and fluidics were used according to the surgical protocol at our institution. The Eight-Chop Technique was selected as the standardized surgical approach in this study to maintain procedural consistency and minimize technique-related variability across cohorts. This study was not designed to compare the Eight-Chop Technique with alternative phacoemulsification approaches.

### 2.4. Intraoperative and Postoperative Outcome Measures

The intraoperative parameters evaluated in this study were operative time (min), phaco time (s), aspiration time (s), cumulative dissipated energy (CDE), irrigation fluid volume (mL), and the incidence of intraoperative complications. Operative time was defined as the interval from creation of the corneal incision to the completion of aspiration of the viscoelastic material. The use of iris retractor hooks was recorded as a binary intraoperative variable (used or not used) after review of the surgical database and operative videos.

The postoperative outcome measures were BCVA, IOP, and CECD at 7 and 19 weeks after surgery. CECD loss at 19 weeks was evaluated as a postoperative corneal endothelial outcome. All surgeries were digitally recorded, and a standardized electronic database was maintained for all cases. Intraoperative complications, including posterior capsule rupture, zonular dialysis, and clinically significant iris trauma, were prospectively recorded in this database. For the present study, both the database entries and the surgical videos were systematically reviewed for all eyes to confirm the presence or absence of intraoperative complications.

### 2.5. Statistical Analysis

Statistical analyses were performed using R software, version 4.5.2 (R Foundation for Statistical Computing, Vienna, Austria). Continuous variables are presented as mean ± standard deviation, and categorical variables are presented as counts and percentages. Preoperative characteristics were compared among the 3 age groups using one-way analysis of variance or the Kruskal–Wallis test for continuous variables and the chi-square test or Fisher’s exact test for categorical variables, as appropriate. The analysis included 169 eyes: 60 eyes in the 70–79-year group, 60 eyes in the 80–89-year group, and 49 eyes in the ≥90-year group. These 169 eyes were contributed by 147 patients; 22 patients contributed both eyes and 125 patients contributed one eye. To account for within-patient correlation between fellow eyes, linear mixed-effects models [15] were fitted with age group as a fixed effect and patient as a random intercept. Operative time, phacoemulsification time, aspiration time, CDE, and irrigation fluid volume were entered as dependent variables in separate models. Although the same eligibility criteria were applied across age groups, the retrospective observational design could not eliminate residual confounding due to differences in nuclear hardness, pupillary status, zonular condition, ocular comorbidities, and other measured or unmeasured clinical characteristics. For postoperative outcomes, linear mixed-effects models were used to compare BCVA, CECD, and percentage CECD loss at 19 weeks among the 3 age groups, again specifying age group as a fixed effect and patient as a random effect. As a sensitivity analysis, the operative-time model was repeated after excluding all eyes in which iris retractor hooks were used in any age group, using the same linear mixed-effects model with age group as a fixed effect and patient as a random effect. This analysis was performed to assess whether the between-group differences in operative time persisted after excluding eyes requiring additional pupillary-dilation maneuvers. Estimated marginal means and pairwise between-group mean differences were obtained using the emmeans package. For pairwise comparisons, 95% confidence intervals and *p* values were adjusted using Tukey’s method. Model-based F statistics and corresponding *p* values for the fixed effect of age group were obtained using Satterthwaite’s approximation for denominator degrees of freedom, as implemented in the lmerTest package. A *p* value < 0.05 was considered statistically significant, and all tests were two-tailed. Because of the retrospective observational design, *p* values and confidence intervals are presented as measures of association between age groups and should not be interpreted as evidence of causality.

### 2.6. GenAI Statement

During the preparation of this study, the author used an AI-based language assistant (Perplexity Computer; Perplexity AI, San Francisco, CA, USA; https://www.perplexity.ai/, accessed on 30 August 2026) to help refine the wording of this manuscript and to draft responses to peer-review comments. The author has reviewed and edited all AI-assisted text and takes full responsibility for the scientific content, analyses, and conclusions of this publication.

## 3. Results

### 3.1. Baseline Characteristics

A total of 169 eyes from 147 patients were included in the present analysis and stratified into three age groups: 70–79 years (60 eyes), 80–89 years (60 eyes), and ≥90 years (49 eyes). Of the 169 eyes included in the present analysis, 27 eyes (16.0%) overlapped with cases included in previous reports from our institution. Because some eyes were included in more than one previous report, these overlaps corresponded to 34 matched records. Baseline demographic and preoperative characteristics are presented in Table 1. Emery nuclear grade distributions differed significantly among the age groups (*p* < 0.001). Grade II cataracts were observed in 54 eyes (90%) in the 70–79-year group, 49 eyes (82%) in the 80–89-year group, and 30 eyes (61%) in the ≥90-year group. In contrast, grade IV cataracts were more frequent in the ≥90-year group (14 eyes, 29%) than in the 70–79-year group (2 eyes, 3.3%) and 80–89-year group (3 eyes, 5.0%). Preoperative BCVA was worse in the nonagenarian cohort than in the younger reference cohorts (*p* < 0.001), whereas preoperative IOP and CECD did not differ significantly among the cohorts (Table 1). Despite application of the same eligibility criteria across age groups, baseline clinical characteristics and surgical complexity differed among the cohorts. Across the 70–79-year, 80–89-year, and ≥90-year groups, respectively, glaucoma was present in 6, 6, and 8 eyes; preoperative topical antiglaucoma medications were used in 3, 2, and 2 eyes; age-related macular degeneration was present in 0, 1, and 6 eyes; obvious zonular weakness was present in 1, 1, and 2 eyes; white cataract was present in 0, 0, and 3 eyes; pseudoexfoliation was present in 1, 0, and 1 eyes; and poor pupillary dilation was present in 6, 8, and 15 eyes. Iris retractor hooks were used in 3, 7, and 13 eyes, respectively. These eyes were retained in the analysis. These differences in baseline clinical complexity reflected the real-world case mix of the age-stratified cohorts and were considered when interpreting subsequent between-cohort comparisons.

### 3.2. Intraoperative Parameters

Intraoperative parameters differed among the three cohorts and were highest in the nonagenarian cohort (Table 2). Mean operative time was 6.0 ± 2.5 min in the 70–79-year cohort, 6.7 ± 3.7 min in the 80–89-year cohort, and 10.7 ± 6.1 min in the nonagenarian cohort (*p* < 0.001; linear mixed-effects model). Phacoemulsification time was 17.6 ± 7.6, 20.3 ± 8.3, and 30.2 ± 12.0 s, respectively, and aspiration time was 76.8 ± 27.7, 84.6 ± 35.6, and 110.2 ± 35.0 s, respectively (all *p* < 0.001). Cumulative dissipated energy was 6.67 ± 2.51, 7.68 ± 3.45, and 10.72 ± 3.76, respectively, and irrigation fluid volume was 30.5 ± 11.9, 33.8 ± 15.6, and 45.2 ± 14.9 mL, respectively (all *p* < 0.001). Model-based pairwise comparisons accounting for within-patient correlation are presented in Supplementary Table S1. Compared with the 70–79-year cohort, the ≥90-year cohort had longer operative time (mean difference, 5.00 min; 95% CI, 2.91 to 7.08), phacoemulsification time (12.80 s; 95% CI, 8.14 to 17.47), and aspiration time (35.67 s; 95% CI, 18.01 to 53.33), as well as higher CDE (mean difference, 4.03; 95% CI, 2.38 to 5.68) and irrigation fluid volume (15.59 mL; 95% CI, 8.03 to 23.15); all Tukey-adjusted *p* values were <0.0001. The ≥90-year cohort also had higher values than the 80–89-year cohort for all five intraoperative parameters (all Tukey-adjusted *p* ≤ 0.0008; Supplementary Table S1). Despite these increases in operative time and energy use, no major intraoperative complications, such as posterior capsule rupture, zonular dialysis, or clinically significant iris trauma, were identified in any of the age groups on review of the surgical database and operative videos. Iris retractor hooks were used in 3 eyes in the 70–79-year group, 7 eyes in the 80–89-year group, and 13 eyes in the ≥90-year group. The results of the sensitivity analysis are presented in Table 3. In the sensitivity analysis excluding all 23 eyes requiring iris retractor hooks, 57, 53, and 36 eyes remained in the 70–79-year, 80–89-year, and ≥90-year groups, respectively. Mean operative time was 5.61 ± 1.88 min, 5.88 ± 2.46 min, and 7.65 ± 2.55 min, respectively. The overall age-group effect remained significant in the linear mixed-effects model (F = 8.29, denominator df = 116.96, *p* < 0.001). In the linear mixed-effects model, operative time was estimated to be 2.18 min longer in the ≥90-year group than in the 70–79-year group (95% CI, 0.87 to 3.50; Tukey-adjusted *p* < 0.001) and 1.92 min longer than in the 80–89-year group (95% CI, 0.60 to 3.25; Tukey-adjusted *p* = 0.002). Prolonged operative time may reflect greater case complexity, including small pupils requiring iris retractor hooks, concomitant ocular conditions, and additional intraoperative maneuvers, rather than age alone. Because excluding cases with iris retractor hooks does not remove other differences in baseline clinical and surgical characteristics, these findings should be interpreted as observational associations rather than as an independent effect of age.

### 3.3. Postoperative Outcomes

Postoperative BCVA improved relative to preoperative values in all age groups (Table 4). At postoperative week 7, mean BCVA was −0.046 ± 0.064, −0.016 ± 0.106, and 0.121 ± 0.344 logMAR in the 70–79-year, 80–89-year, and ≥90-year groups, respectively. At postoperative week 19, mean BCVA was −0.047 ± 0.062, −0.013 ± 0.110, and 0.096 ± 0.364 logMAR, respectively, and differed among the age groups (*p* < 0.001), with the nonagenarian cohort showing the highest mean logMAR value.

**Figure 1:**
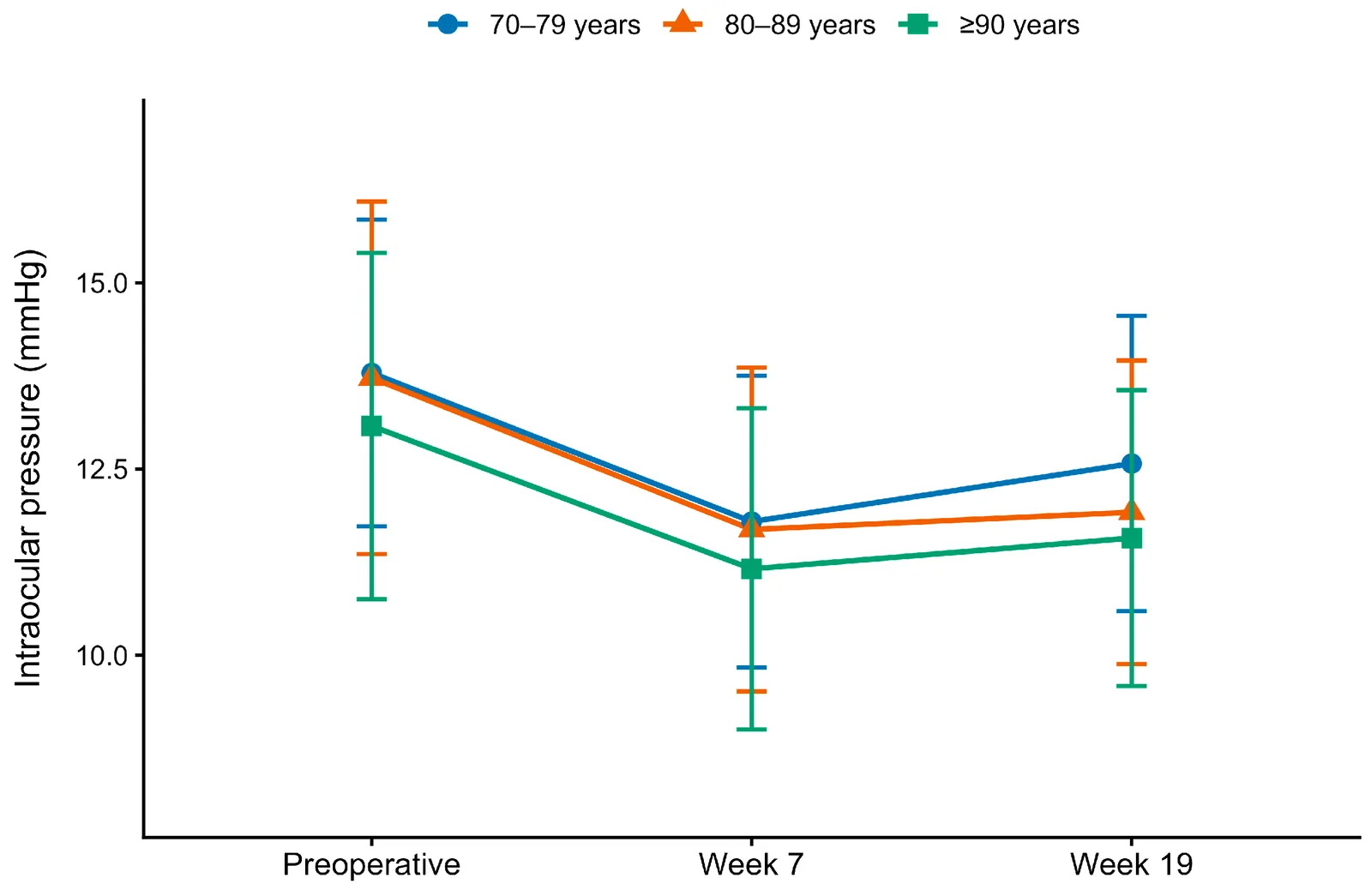
Changes in intraocular pressure from preoperative baseline to 19 weeks after surgery according to age group. Mean intraocular pressure is shown for the 70–79-year, 80–89-year, and ≥90-year groups at the preoperative assessment and at postoperative weeks 7 and 19. Error bars indicate standard deviations.

IOP changes are shown in Figure 1. IOP remained stable throughout the follow-up in all groups. Mean IOP at postoperative weeks 7 and 19 was 11.8 ± 2.0 and 12.6 ± 2.0 mmHg in the 70–79-year group, 11.7 ± 2.2 and 11.9 ± 2.0 mmHg in the 80–89-year group, and 11.2 ± 2.2 and 11.6 ± 2.0 mmHg in the ≥90-year group, with no clinically relevant elevations compared with preoperative values.

**Figure 2:**
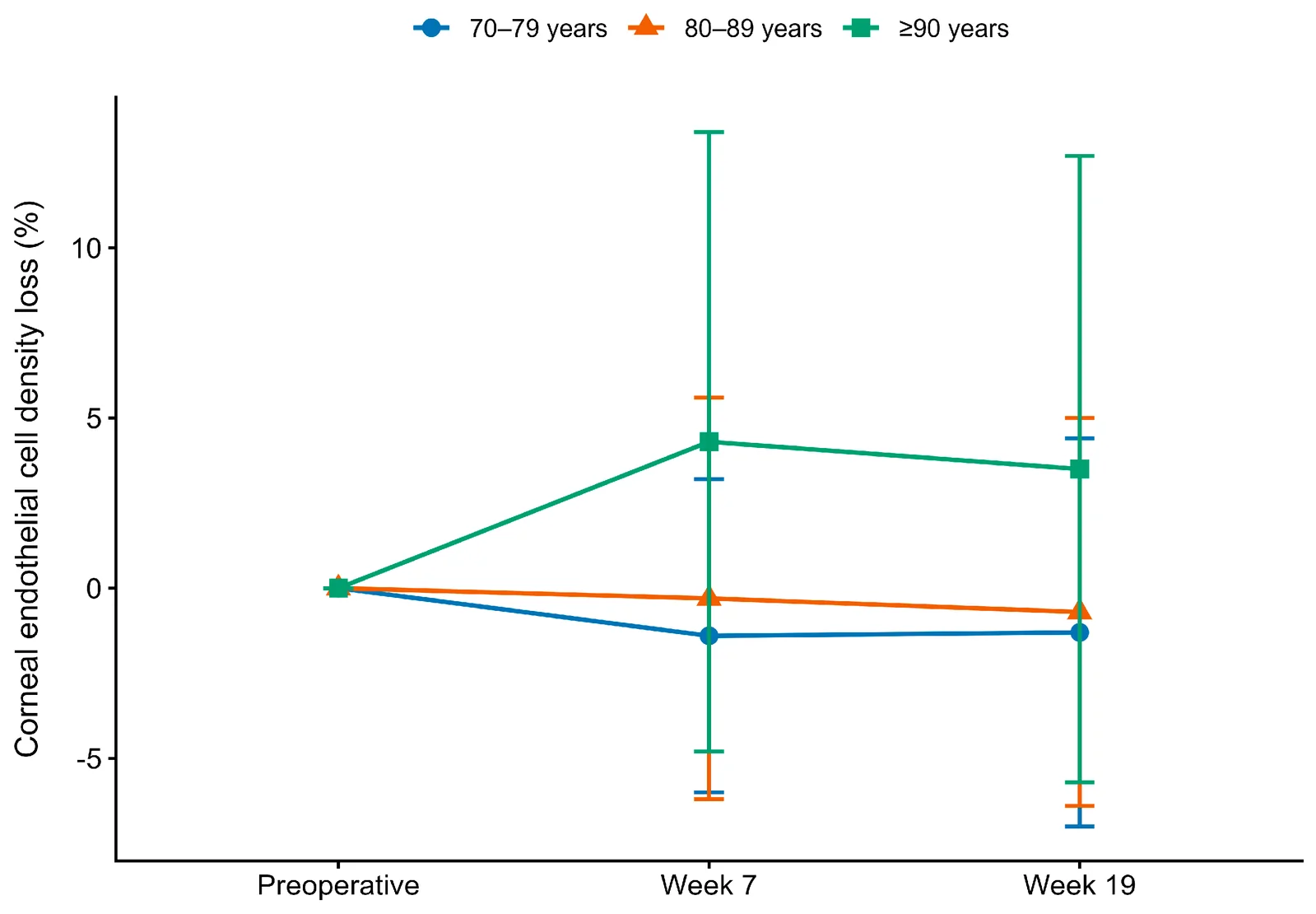
CECD loss at postoperative weeks 7 and 19 according to age group. Points represent mean CECD loss, and error bars represent standard deviations. CECD, corneal endothelial cell density.

Corneal endothelial outcomes, including CECD loss, are shown in Table 4 and Figure 2. Mean CECD at postoperative weeks 7 and 19 was 2615.1 ± 236.9 and 2615.9 ± 261.7 cells/mm^2^ in the 70–79-year group, 2573.7 ± 321.0 and 2594.4 ± 364.1 cells/mm^2^ in the 80–89-year group, and 2474.0 ± 328.1 and 2483.4 ± 313.8 cells/mm^2^ in the ≥90-year group, respectively. At postoperative week 19, CECD did not differ significantly among the cohorts in the linear mixed-effects model (*p* = 0.104). Mean CECD loss at postoperative week 19 differed significantly among the three age groups (*p* = 0.001), with values of −1.3 ± 5.7%, −0.7 ± 5.7%, and 3.5 ± 9.2% in the 70–79-year, 80–89-year, and ≥90-year cohorts, respectively. These findings represent descriptive outcomes in cohorts with differing baseline clinical and surgical complexity.

### 3.4. Complications

No major intraoperative complications, including posterior capsule rupture, dropped nucleus, zonular dialysis, or clinically significant iris trauma, were identified in any study group.

## 4. Discussion

### 4.1. Principal Findings

In this study, we described intraoperative and postoperative outcomes of the Eight-Chop Technique in elderly patients, including a real-world nonagenarian cohort. Compared with the younger elderly reference cohorts, the nonagenarian cohort had worse preoperative BCVA and a greater burden of ocular comorbidities and factors associated with surgical complexity. Accordingly, the intraoperative and postoperative findings should be interpreted as descriptive differences in real-world case mix rather than effects attributable to age alone. In the real-world nonagenarian cohort, operative time, phaco time, aspiration time, CDE, and irrigation fluid volume were higher than in the younger elderly reference cohorts; mean CECD loss at 19 weeks was 3.5 ± 9.2% among eyes with available paired measurements, and postoperative visual acuity improved relative to baseline in all cohorts. Importantly, no serious intraoperative complications were identified in any cohort after review of the surgical database and operative videos; specifically, no posterior capsule rupture, zonular dialysis, or clinically significant iris trauma occurred. This observation does not establish comparative safety or safety attributable to the Eight-Chop Technique. In a sensitivity analysis excluding all eyes requiring iris retractor hooks, operative time remained significantly longer in the ≥90-year group than in the 70–79-year group (mean difference, 2.18 min; 95% CI, 0.87–3.50; Tukey-adjusted *p* < 0.001) and the 80–89-year group (mean difference, 1.92 min; 95% CI, 0.60–3.25; Tukey-adjusted *p* = 0.002). This finding indicates that the longer operative time in the ≥90-year cohort was not explained solely by iris retractor hook use. However, because other differences in baseline clinical and surgical complexity remained between the cohorts, the result should not be interpreted as an independent causal effect of age on operative time.

### 4.2. Comparison with Previous Studies

Previous reports have highlighted the challenges of cataract surgery in very elderly patients, including higher nuclear hardness, zonular weakness, and increased risk of intraoperative and postoperative complications [16,17,18]. Many studies have reported corneal endothelial cell loss in the range of roughly 5–10% or more after phacoemulsification in routine elderly populations, with higher losses in dense nuclei and in eyes with longer phaco time and higher CDE [7,8,9,10,19,20,21,22,23,24,25,26]. The observed mean CECD loss at 19 weeks was 3.5 ± 9.2% in the nonagenarian cohort. This estimate should be interpreted cautiously because of the relatively large interindividual variability, incomplete follow-up, the retrospective single-center design, and the absence of a contemporaneous comparator technique. However, this finding should not be interpreted as evidence that the Eight-Chop Technique independently prevented endothelial injury, because the nonagenarian cohort differed from the younger reference cohorts in baseline clinical and surgical complexity. Although intraoperative parameters differed across age groups, the observational design does not permit attribution of postoperative endothelial outcomes to a specific surgical feature. Further prospective studies with contemporaneous comparator techniques are needed to determine whether specific nucleus-division strategies influence endothelial outcomes in elderly patients.

Visual outcomes in nonagenarian patients have been reported to be generally favorable when systemic status and ocular comorbidities are appropriately managed [4,6,27,28], although some studies note more modest gains compared with younger cohorts because of pre-existing macular or optic nerve disease [29,30]. The present results align with these observations: compared with the younger reference cohorts, the ≥90-year group had worse preoperative BCVA, a greater burden of ocular comorbidities, and worse postoperative BCVA. Although postoperative visual acuity improved from baseline in all cohorts, these findings should not be interpreted as equivalent visual outcomes across age groups, because the cohorts differed in baseline clinical complexity and real-world case mix. This finding reinforces the value of cataract surgery for quality of life in the nonagenarians, while also emphasizing that expectations should be individualized based on coexisting ocular pathologies.

### 4.3. Clinical Implications

The findings of this study provide descriptive information that may assist surgical planning for nonagenarian cataract patients. In this real-world cohort, the nonagenarian group included eyes with greater baseline clinical complexity than the younger reference cohorts. The longer operative time and greater phacoemulsification-related requirements observed in the nonagenarian group should be interpreted in the context of this greater case complexity, rather than as effects attributable to age alone. When planning cataract surgery for nonagenarian patients, surgeons should anticipate the potential for longer operative time, provide individualized preoperative counseling regarding case complexity and visual potential, assess corneal endothelial reserve, and consider appropriate pupil-management devices when clinically indicated, particularly in eyes with poor pupillary dilation. Surgeons should also consider potential dense nuclei, zonular weakness, ocular comorbidities, and reduced postoperative visual potential. The operative time sensitivity analysis showed that mean operative time in the nonagenarian cohort was lower after exclusion of eyes in which iris retractor hooks were used. However, operative time remained longer than in the younger reference cohorts after exclusion, and other differences in clinical and surgical complexity remained. Therefore, this analysis does not demonstrate an independent effect of age on operative time. The mean CECD loss at 19 weeks in the nonagenarian cohort was 3.5 ± 9.2% among eyes with available paired measurements. However, because this study had no contemporaneous comparator technique and the cohorts differed in baseline clinical and surgical complexity, these findings do not establish an endothelial protective effect of the Eight-Chop Technique.

Importantly, no serious intraoperative complications were identified in any cohort after review of the surgical database and operative videos; specifically, no posterior capsule rupture, zonular dialysis, or clinically significant iris trauma occurred. However, this observation does not establish the comparative safety or safety advantage of the Eight-Chop Technique. Because this study did not include a contemporaneous cohort treated with another nucleus-division technique, the present data do not establish the comparative safety, efficacy, endothelial protection, or superiority of the Eight-Chop Technique.

### 4.4. Limitations and Future Directions

This study has several limitations. First, it was conducted at a single center, and all surgeries were performed by a single experienced surgeon using a single surgical technique. Therefore, the findings may not be generalizable to other clinical settings, surgeons with different levels of experience, or alternative phacoemulsification techniques. Second, the retrospective observational design and the absence of a contemporaneous comparator technique preclude causal inference regarding age-group differences or the independent effects of the Eight-Chop Technique. Accordingly, the present findings should be interpreted as descriptive and hypothesis-generating rather than as a definitive comparison of age effects. Third, the same prespecified inclusion and exclusion criteria were applied across all age groups. In response to the Editor’s request, the 70–79-year and 80–89-year reference cohorts were reselected by random sampling of 60 eligible eyes per group from cases treated during the same study period as the ≥90-year cohort. Nevertheless, residual differences in nuclear hardness, pupillary status, zonular condition, ocular comorbidities, and other measured or unmeasured clinical characteristics remained among the age groups. Therefore, between-group comparisons should be interpreted as observational associations and do not establish an independent causal effect of age. The present study was not designed as an age-matched control study; rather, it compared age-stratified cohorts reflecting real-world case mix. Accordingly, observed differences in operative time, pupil-management requirements, and postoperative outcomes may reflect residual differences in case mix and surgical complexity between cohorts, rather than age alone. Moreover, CECD was measured using a single noncontact specular microscope, and although the near-zero mean percentage changes in the younger elderly groups are broadly consistent with previous Eight-Chop studies from the same center, some degree of measurement variability cannot be excluded and should be considered when interpreting the absolute magnitude of endothelial cell loss. Minor or transient postoperative events, including early postoperative intraocular pressure elevation and Descemet membrane folds, were not systematically assessed, and detailed event-level records were unavailable for this retrospective analysis; therefore, their incidence and potential associations with age or surgical complexity could not be evaluated reliably. In addition, follow-up of 19 weeks was sufficient to assess early and mid-term outcomes, but longer-term changes in CECD and visual function were not evaluated. Finally, the ≥90-year group included fewer eyes than the younger groups, and some postoperative outcome data were missing during follow-up, which may have reduced precision. At postoperative week 19, outcome data were available for 32 of the initial 49 eyes in the nonagenarian cohort. The missing data resulted from non-attendance at scheduled follow-up visits. Because this was a retrospective study, the specific reasons for non-attendance, including death, reduced mobility, deterioration in general health, transfer of care, or other factors, were not systematically recorded. Therefore, a reliable breakdown of the reasons for loss to follow-up was not available. Attrition bias or survivorship bias in the assessment of late BCVA and corneal endothelial outcomes cannot be excluded, because patients who returned for follow-up may have differed from those who did not return in general health, ocular status, or postoperative outcomes. Accordingly, the 19-week results should be interpreted as descriptive findings based on the available follow-up data and may not fully represent outcomes in the entire original nonagenarian cohort. Future prospective multicenter studies with larger nonagenarian cohorts and comparator techniques will be important to confirm the generalizability of these findings.

## 5. Conclusions

The present study describes intraoperative and postoperative outcomes of the Eight-Chop Technique in elderly patients, including a real-world nonagenarian cohort with greater baseline clinical and surgical complexity than the younger reference cohorts. Postoperative visual acuity improved relative to preoperative values in all cohorts, and mean CECD loss at 19 weeks was 3.5 ± 9.2% in the nonagenarian cohort among eyes with available paired measurements. These descriptive findings do not establish that the Eight-Chop Technique independently minimizes endothelial stress.

Within this carefully managed cohort, the findings suggest that the Eight-Chop Technique was clinically feasible for cataract surgery in nonagenarians. However, this retrospective, single-center, single-surgeon study did not include a contemporaneous comparator technique, and differences in real-world case mix and baseline clinical characteristics resulted in greater clinical and surgical complexity in the nonagenarian cohort than in the younger reference cohorts. Therefore, the results should be interpreted as descriptive and hypothesis-generating and do not establish comparative safety, efficacy, endothelial protection, or superiority of the Eight-Chop Technique. Larger prospective multicenter studies involving multiple surgeons, with standardized assessment and adjustment for baseline clinical and surgical complexity, contemporaneous comparator techniques, and longer follow-up, are needed to validate these observations and assess their generalizability.

## Figures and Tables

**Table 1:** Preoperative characteristics according to age group.

Characteristic	70–79 Years *n* = 60	80–89 Years *n* = 60	≥90 Years *n* = 49	*p-*Value
Patients, *n*	60	60	27	
Patients contributing one eye/both eyes, *n*	60/0	60/0	5/22	
Age, years	74.8 ± 2.5	82.6 ± 2.4	91.9 ± 2.0	<0.001
Sex				0.479
Female	33 (55%)	38 (63%)	32 (65%)	
Male	27 (45%)	22 (37%)	17 (35%)	
Operated eye				0.823
L	28 (47%)	30 (50%)	26 (53%)	
R	32 (53%)	30 (50%)	23 (47%)	
Emery nuclear grade				<0.001
II	54 (90%)	49 (82%)	30 (61%)	
III	4 (6.7%)	8 (13%)	5 (10%)	
IV	2 (3.3%)	3 (5.0%)	14 (29%)	
Preoperative BCVA, logMAR	0.191 ± 0.335	0.199 ± 0.285	0.482 ± 0.555	<0.001
Preoperative IOP, mmHg	13.8 ± 2.1	13.7 ± 2.4	13.1 ± 2.3	0.265
Preoperative CECD, cells/mm^2^	2588.1 ± 237.9	2566.3 ± 336.2	2585.3 ± 267.8	0.968
Preoperative CV, %	39.3 ± 5.8	40.9 ± 6.1	40.8 ± 5.6	0.132
Preoperative PHC, %	43.3 ± 7.4	44.3 ± 7.2	45.5 ± 6.9	0.335
Preoperative CCT, μm	531.1 ± 39.0	528.6 ± 31.7	522.0 ± 30.2	0.251

Values are presented as mean ± standard deviation or *n* (%). *p* values for continuous variables were calculated using the Kruskal–Wallis test, and *p* values for categorical variables were calculated using Fisher’s exact test. BCVA, best-corrected visual acuity; IOP, intraocular pressure; CECD, corneal endothelial cell density; CV, coefficient of variation in cell size; PHC, percentage of hexagonal cells; CCT, central corneal thickness.

**Table 2:** Intraoperative parameters by age group.

Intraoperative Parameter	70–79 Years *n* = 60	80–89 Years *n* = 60	≥90 Years *n* = 49	*p-*Value
Operative time, min	6.0 ± 2.5	6.7 ± 3.7	10.7 ± 6.1	<0.001
Phacoemulsification time, s	17.6 ± 7.6	20.3 ± 8.3	30.2 ± 12.0	<0.001
Aspiration time, s	76.8 ± 27.7	84.6 ± 35.6	110.2 ± 35.0	<0.001
Cumulative dissipated energy	6.67 ± 2.51	7.68 ± 3.45	10.72 ± 3.76	<0.001
Fluid volume, mL	30.5 ± 11.9	33.8 ± 15.6	45.2 ± 14.9	<0.001
Iris retractor hooks use	3 (5.0%)	7 (12%)	13 (27%)	0.005

Values are presented as mean ± standard deviation or *n* (%). For continuous intraoperative parameters, overall age-group *p* values were derived from separate linear mixed-effects models with age group as a fixed effect and patient ID as a random intercept to account for correlation between fellow eyes. Model-based pairwise comparisons, including estimated mean differences, Tukey-adjusted 95% confidence intervals, and Tukey-adjusted *p* values, are presented in Supplementary Table S1. The *p* value for iris retractor hook use was calculated using Fisher’s exact test.

**Table 3:** Sensitivity analysis of operative time after exclusion of eyes requiring iris retractor hooks.

Age Group	Eyes Analyzed	Patients	Operative Time, min	Estimated Marginal Mean, min (95% CI)
70–79 years	57	57	5.61 ± 1.88	5.61 (5.02–6.21)
80–89 years	53	53	5.88 ± 2.46	5.88 (5.26–6.49)
≥90 years	36	21	7.65 ± 2.55	7.80 (6.88–8.72)
			Mean difference, min (95% CI)	*p*-Value
Overall age-group effect			F = 8.29; denominator df = 116.96	<0.001
≥90 vs. 70–79 years			2.18 (0.87–3.50)	<0.001
≥90 vs. 80–89 years			1.92 (0.60–3.25)	0.002
70–79 vs. 80–89 years			−0.26 (−1.29 to 0.76)	0.818

Values are presented as mean ± standard deviation unless otherwise indicated. Estimated marginal means and pairwise comparisons were obtained from a linear mixed-effects model with age group as a fixed effect and patient as a random effect. Eyes requiring iris retractor hooks were excluded. The overall age-group effect was tested using Satterthwaite’s approximation. Pairwise *p* values and 95% confidence intervals were Tukey adjusted. The number of patients reflects the patients contributing the analyzed eyes after exclusion of eyes requiring iris retractor hooks.

**Table 4:** Postoperative visual, intraocular pressure, and corneal endothelial outcomes by age group.

Postoperative Outcome	70–79 Years *n* = 60	80–89 Years *n* = 60	≥90 Years *n* = 49	*p*-Value
BCVA at postoperative week 7, logMAR	−0.046 ± 0.064	−0.016 ± 0.106	0.121 ± 0.344	<0.001
BCVA at postoperative week 19, logMAR	−0.047 ± 0.062	−0.013 ± 0.110	0.096 ± 0.364	<0.001
IOP at postoperative week 7, mmHg	11.8 ± 2.0	11.7 ± 2.2	11.2 ± 2.2	0.518
IOP at postoperative week 19, mmHg	12.6 ± 2.0	11.9 ± 2.0	11.6 ± 2.0	0.102
CECD at postoperative week 7, cells/mm^2^	2615.1 ± 236.9	2573.7 ± 321.0	2474.0 ± 328.1	0.043
CECD at postoperative week 19, cells/mm^2^	2615.9 ± 261.7	2594.4 ± 364.1	2483.4 ± 313.8	0.104
CECD loss at postoperative week 7, %	−1.4 ± 4.6	−0.3 ± 5.9	4.3 ± 9.1	<0.001
CECD loss at postoperative week 19, %	−1.3 ± 5.7	−0.7 ± 5.7	3.5 ± 9.2	0.001
CV at postoperative week 7, %	39.3 ± 4.5	38.7 ± 5.4	39.2 ± 4.4	0.399
CV at postoperative week 19, %	37.3 ± 5.2	36.7 ± 5.7	38.9 ± 4.7	0.082
PHC at postoperative week 7, %	44.5 ± 5.7	45.1 ± 6.4	44.1 ± 6.1	0.771
PHC at postoperative week 19, %	46.6 ± 6.1	47.4 ± 7.0	45.1 ± 4.6	0.243
CCT at postoperative week 7, μm	535.0 ± 38.8	530.7 ± 37.1	522.6 ± 32.4	0.329
CCT at postoperative week 19, μm	536.3 ± 37.1	525.3 ± 34.0	525.9 ± 32.6	0.386

Values are presented as mean ± standard deviation. *p* values were derived from linear mixed-effects models with age group as a fixed effect and patient as a random effect to account for correlation between fellow eyes. Numbers of eyes with available data varied by outcome and follow-up time; at postoperative week 19, BCVA and/or CECD outcome data were available for 32 of 49 eyes in the ≥90-year group. BCVA, best-corrected visual acuity; logMAR, logarithm of the minimum angle of resolution; IOP, intraocular pressure; CECD, corneal endothelial cell density; CV, coefficient of variation in cell size; PHC, percentage of hexagonal cells; CCT, central corneal thickness.

## Data Availability

The original contributions presented in this study are included in the article/Supplementary Materials. Further inquiries can be directed to the corresponding author.

## Supplementary Materials

The following supporting information can be downloaded at https://www.mdpi.com/article/10.3390/jcm15176737/s1. Supplementary Dataset: Anonymized original dataset for the EightChop_nonagenarian study (Nonagenarian_data02.xlsx). Supplementary Table S1: Model-based pairwise comparisons of intraoperative parameters among age groups (Supplementary_Table_S1.docx).

## Abbreviations

The following abbreviations are used in this manuscript:

BCVA	Best corrected visual acuity
CCT	Central corneal thickness
CDE	Cumulative dissipated energy
CECD	Corneal endothelial cell density
CV	Coefficient of variation in cell size
IOP	Intraocular pressure
logMAR	Logarithm of the minimum angle of resolution
PHC	Percentage of hexagonal cells
