# Supplementary material for: The Eight-Chop Technique in Nonagenarian Patients: Real-World Intraoperative and Postoperative Outcomes
Source: J Clin Med. 2026 Aug 30;15(17):6737. doi: 10.3390/jcm15176737 (PMC13565972; doi:10.3390/jcm15176737)
Supplement: Supplementary file 1 [file jcm-15-06737-s001.zip › Supplementary_Table_S1.pdf]

Supplementary Table S1

Model-Based Pairwise Comparisons of Intraoperative Outcomes Across Age Groups

Outcome	Comparison	Estimated mean difference	95% CI	Tukey-adjusted P value	Eyes, n	Patients, n
Operative time, min	80–89 years − 70–79 years	0.75	-1.03 to 2.52	0.5817	169	147
Operative time, min	≥90 years − 70–79 years	5.00	2.91 to 7.08	<0.0001	169	147
Operative time, min	≥90 years − 80–89 years	4.25	2.16 to 6.34	<0.0001	169	147
Phacoemulsification time, s	80–89 years − 70–79 years	2.66	-1.28 to 6.60	0.2498	169	147
Phacoemulsification time, s	≥90 years − 70–79 years	12.80	8.14 to 17.47	<0.0001	169	147
Phacoemulsification time, s	≥90 years − 80–89 years	10.14	5.47 to 14.80	<0.0001	169	147
Aspiration time, s	80–89 years − 70–79 years	7.83	-6.45 to 22.12	0.3985	169	147
Aspiration time, s	≥90 years − 70–79 years	35.67	18.01 to 53.33	<0.0001	169	147
Aspiration time, s	≥90 years − 80–89 years	27.84	10.18 to 45.50	0.0008	169	147
Cumulative dissipated energy	80–89 years − 70–79 years	1.00	-0.38 to 2.39	0.2043	169	147
Cumulative dissipated energy	≥90 years − 70–79 years	4.03	2.38 to 5.68	<0.0001	169	147
Cumulative dissipated energy	≥90 years − 80–89 years	3.02	1.38 to 4.67	<0.0001	169	147
Fluid volume, mL	80–89 years − 70–79 years	3.27	-2.90 to 9.44	0.4238	169	147
Fluid volume, mL	≥90 years − 70–79 years	15.59	8.03 to 23.15	<0.0001	169	147
Fluid volume, mL	≥90 years − 80–89 years	12.32	4.77 to 19.88	0.0005	169	147

Note. Separate linear mixed-effects models were fitted for each outcome, with age group as a fixed effect and patient ID as a random intercept to account for within-patient correlation. Mean differences are calculated as the higher age group minus the lower age

group. Confidence intervals and P values were adjusted using Tukey’s method for the three pairwise comparisons within each outcome. CI = confidence interval.
